# Comprehensive genome assembly reveals genetic diversity and carcass consumption insights in critically endangered Asian king vultures

**DOI:** 10.1038/s41598-024-59990-9

**Published:** 2024-04-24

**Authors:** Wannapol Buthasane, Vorasuk Shotelersuk, Wanna Chetruengchai, Chalurmpon Srichomthong, Adjima Assawapitaksakul, Sithichoke Tangphatsornruang, Wirulda Pootakham, Chutima Sonthirod, Sissades Tongsima, Pongsakorn Wangkumhang, Alisa Wilantho, Ampika Thongphakdee, Saowaphang Sanannu, Chaianan Poksawat, Tarasak Nipanunt, Chaiyan Kasorndorkbua, Klaus-Peter Koepfli, Budhan S. Pukazhenthi, Prapat Suriyaphol, Thidathip Wongsurawat, Piroon Jenjaroenpun, Gunnaporn Suriyaphol

**Affiliations:** 1 Biochemistry Unit, Department of Physiology, Faculty of Veterinary Science, Chulalongkorn University, Bangkok, 10330 Thailand; 2 Center of Excellence for Medical Genomics, Department of Pediatrics, Faculty of Medicine, Chulalongkorn University, Henri Dunant Road, Pathumwan, Bangkok, 10330 Thailand; 3 Excellence Center for Genomics and Precision Medicine, King Chulalongkorn Memorial Hospital, The Thai Red Cross Society, Bangkok, 10330 Thailand; 4 National Center for Genetic Engineering and Biotechnology (BIOTEC), National Science and Technology Development Agency, Pathum Thani, 12120 Thailand; 5 Animal Conservation and Research Institute, The Zoological Park Organization of Thailand under the Royal Patronage of H.M. The King, Bangkok, 10300 Thailand; 6 Huai Kha Khaeng Wildlife Breeding Center, Department of National Parks, Wildlife and Plant Conservation, Uthai Thani, 61160 Thailand; 7 Laboratory of Raptor Research and Conservation Medicine, Department of Pathology, Faculty of Veterinary Medicine, Kasetsart University, Bangkok, 10900 Thailand; 8 Smithsonian–Mason School of Conservation, George Mason University, Front Royal, VA 22630 USA; 9 Center for Species Survival, Smithsonian Conservation Biology Institute, National Zoological Park, Front Royal, VA 22630 USA; 10 Division of Medical Bioinformatics, Department of Research and Development, Faculty of Medicine Siriraj Hospital, Mahidol University, Bangkok, 10700 Thailand

**Keywords:** Evolution, Genetics, Molecular biology, Systems biology, Zoology

## Abstract

The Asian king vulture (AKV), a vital forest scavenger, is facing globally critical endangerment. This study aimed to construct a reference genome to unveil the mechanisms underlying its scavenger abilities and to assess the genetic relatedness of the captive population in Thailand. A reference genome of a female AKV was assembled from sequencing reads obtained from both PacBio long-read and MGI short-read sequencing platforms. Comparative genomics with New World vultures (NWVs) and other birds in the Family Accipitridae revealed unique gene families in AKV associated with retroviral genome integration and feather keratin, contrasting with NWVs’ genes related to olfactory reception. Expanded gene families in AKV were linked to inflammatory response, iron regulation and spermatogenesis. Positively selected genes included those associated with anti-apoptosis, immune response and muscle cell development, shedding light on adaptations for carcass consumption and high-altitude soaring. Using restriction site-associated DNA sequencing (RADseq)-based genome-wide single nucleotide polymorphisms (SNPs), genetic relatedness and inbreeding status of five captive AKVs were determined, revealing high genomic inbreeding in two females. In conclusion, the AKV reference genome was established, providing insights into its unique characteristics. Additionally, the potential of RADseq-based genome-wide SNPs for selecting AKV breeders was demonstrated.

## Introduction

The Asian king vulture (AKV), also known as the red-headed vulture (*Sarcogyps calvus*), belongs to the Old World vulture (OWV) group within the order Accipitriformes and family Accipitridae. This species is classified as critically endangered (CR), according to the International Union for Conservation of Nature (IUCN) Red List of Threatened Species^1^. Additionally, it is listed in Appendix II of the Convention on International Trade in Endangered Species of Wild Fauna and Flora (CITES)^2^. The AKV is also considered a protected wild animal under Thailand’s Wild Animal Conservation and Protection Act, B.E. 2562 (2019)^3^. Vultures are a group of birds of prey or diurnal raptors, with a unique dietary preference for carrion. They play a vital role in ecosystem services by efficiently disposing of carcasses that may be contaminated with pathogens and toxins, thereby contributing to the prevention of potential pathogen spillover from wildlife to human communities^4^. OWVs are distributed in Africa, Asia and Europe, while New World vultures (NWVs) can be found in the Americas. Both groups share some common features, such as a bald head, long broad wings and obligatory carrion-feeding behavior, which have evolved convergently^5^. However, OWVs have keen eyesight to locate food, whereas NWVs predominantly rely on a highly developed sense of smell to find carrion^6^.

The AKV population in South Asia experienced a catastrophic decline in the 1990s due to the ingestion of cattle carrion contaminated with diclofenac, a non-steroidal anti-inflammatory drug (NSAID). Diclofenac poses a threat to many OWV species, including white-rumped, slender-billed Eurasian, Himalayan griffon and cinereous vultures^7–9^. In Thailand, the last flock of approximately 30 vultures at Huai Kha Khaeng Wildlife Sanctuary was decimated in 1992 when the vultures consumed a muntjac carcass laced with carbofuran, deliberately placed by poachers to target tigers^10^. Currently, only seven captive individuals exist in Thailand: six at Nakhon Ratchasima Zoo, Zoological Park Organization of Thailand (ZPOT) and one at Huai Kha Khaeng Wildlife Breeding Center, Department of National Park, Wildlife and Plant Conservation (DNP). On March 10, 2023, the Thailand Red-Headed Vulture Project, a collaboration of ZPOT, DNP, Kasetsart University and Seub Nakhasathien Foundation, successfully bred a female AKV chick for the first time in 30 years in Thailand and Asia^11^. Additionally, on February 9, 2024, the first AKV chick was born in the wild at Huai Kha Khaeng Wildlife Sanctuary, its original habitat^12^. The challenge in breeding AKVs may be attributed to limited genetic knowledge of the species, underscoring the urgent need for research in this field.

Genomics provides a wealth of biological information, including genes associated with molecular functions, biological processes and cellular components^13^. Additionally, it offers valuable insights into the relatedness between species^14^. Several studies have demonstrated evolved adaptive mechanisms of carrion-eating species to avoid illness when consuming contaminated carcasses. For instance, positively selected genes (PSGs) related to the immune system, such as the Toll-like receptor signaling pathway, have been identified in hyenas, cinereous vultures and turkey vultures^15,16^. PSGs associated with the digestive system, including the gastric acid secretion pathway, have been reported in Himalayan, turkey and bearded vultures^6,17^. Furthermore, the expansion of β-defensin genes associated with antimicrobial host-defense peptides has been found in the Komodo dragon^18^.

Genome data from one animal serves as the reference for population genomics, facilitating the investigation and comparison of genetic diversity within and between populations. Additionally, it enables the assessment of inbreeding risk and population structure. Instead of conducting costly whole genome resequencing, restriction site-associated DNA sequencing (RADseq) has been employed to provide economically efficient representative sequences of genome-wide single nucleotide polymorphisms (SNPs) in various species, including the Iberian lynx, Burmese roofed turtle and Eld’s deer populations^14,19,20^. Hence, RADseq-based genomic information plays a crucial role in guiding the conservation management of threatened species.

To the best of our knowledge, the genomic background of the AKV has not been extensively studied. Furthermore, insights into AKV genomics, both in terms of comparative and population genomics, remain limited. The objective of the present study was to construct a reference genome of a female AKV using a combination of long-read and short-read sequencing techniques. In addition, we explored the genetic relatedness of five AKVs using genome-wide SNPs generated by RADseq. This approach is proposed as an alternative protocol for evaluating genetic relatedness among captive AKVs in zoos and wildlife breeding centers worldwide, with the aim of facilitating future collaborative efforts in breeder selection and population recovery.

## Results

### De novo genome assembly and annotation

**Figure 1:**
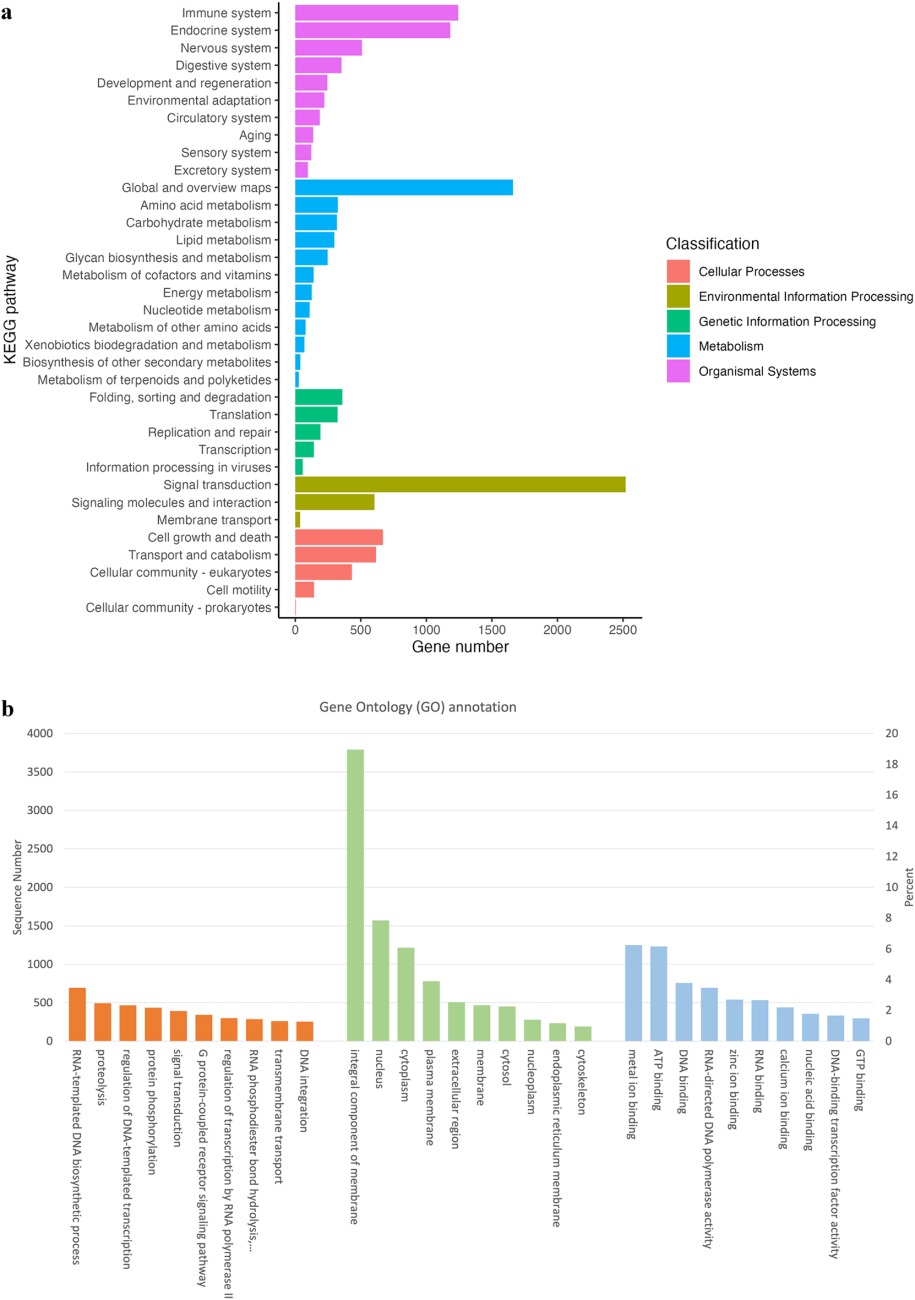
KEGG pathway and Gene Ontology (GO) annotations of the Asian king vulture (AKV) genome. (**a**) Distribution of the number of proteins in different KEGG functional categories annotated using the KEGG Automatic Annotation Server (KAAS). (**b**) GO annotation of AKV.

The whole genome sequencing of a 25-year-old female AKV (RV4F) from Nakhon Ratchasima Zoo was conducted using both PacBio long-read and MGISEQ-2000 short-read platforms. Additionally, the ORG.one project (https://nanoporetech.com/oo) supported two MinION flow cells for Nanopore long-read sequencing. Details on genome assembly, GC content, and Benchmarking Universal Single-Copy Orthologs (BUSCO) assembly scores for two approaches – the PacBio and MGI hybrid sequencing with Purged analysis (PacBio-MGI-Purged), and the Nanopore and MGI hybrid sequencing (Nanopore-MGI) – are presented in Table 1. Due to insufficient Nanopore sequencing data (10 × coverage, total bases 13.5 Gb) for genome assembly, we assembled the reference genome using raw reads sequenced by PacBio (1228 × coverage, total bases 1548.3 Gb) and MGISEQ-2000 only (109 × coverage, total bases 140.6 Gb). Ultimately, we generated a total genome length of 1.29 Gb (1230 × coverage) for the AKV (GenBank accession number: PRJNA827941), with a scaffold N50 of 28.73 Mb, after removing low-quality and duplicated reads. The completeness of the AKV genome was evaluated using 8,338 single-copy orthologs. It achieved 97.4% completeness with 1.1% complete and duplicated BUSCOs, 0.5% corresponding to fragmented BUSCOs and 2.1% indicating missing BUSCOs. Additional details, such as repeat elements, total protein-coding genes, non-coding RNAs, KEGG pathways and Gene Ontology (GO) categories of putative genes for the AKV assembly, can be found in Supplementary Tables 1 and 2 and Fig. 1. We annotated a total of 19,388 protein-coding genes, covering a gene length totality of 236 Mb with an average gene size of 12,148 nucleotides. Out of the annotated genes, 17,444 (89.97%) were functionally annotated through GO, and 13,656 (70.44%) were functionally annotated through KEGG. Exons had a combined length of 31.47 Mb, with an average length of 185 bp, while introns had a total length of 204.07 Mb, with an average length of 1352 bp. The average number of exons per gene was 8.79 and the average number of introns per gene was 7.79. The GC contents in exons and introns were 52.08 and 42.67%, respectively. Sequences with the annotation of endogenous retrovirus were counted (Supplementary Table 3). The proportions of the total endogenous retrovirus sequences in the AKV genome were discovered to be 33%, 16% and 51% for the Env protein, Gag protein and Pol protein, respectively.

**Table 1:** Comparison of genome assembly and quality assessment for Asian king vulture using PacBio and MGI hybrid sequencing with Purged analysis (PacBio-MGI-Purged) versus Nanopore and MGI hybrid sequencing (Nanopore-MGI).

	PacBio-MGI-Purged	Nanopore-MGI
N50 contig/scaffold size (bases)	28,731,705	8,220,899
L50 contig/scaffold number	16	N/A
Assembly size (bases)	1,288,247,345	1,223,963,004
Number of contigs/scaffolds	261	1001
Number of contigs/scaffolds ≥ 1 Kbp	116	N/A
Number of contigs/scaffolds ≥ 10 Kbp	84	N/A
Number of contigs/scaffolds ≥ 10 Mbp	38	N/A
Longest contig/scaffold (bases)	81,203,610	35,362,384
Number of Ns	0	N/A
GC content (%)	42.92	N/A
BUSCO evaluation (% completeness)	97.4	97

N/A, not applicable.

### Phylogenetic analyses based on the nuclear genome

**Figure 2:**
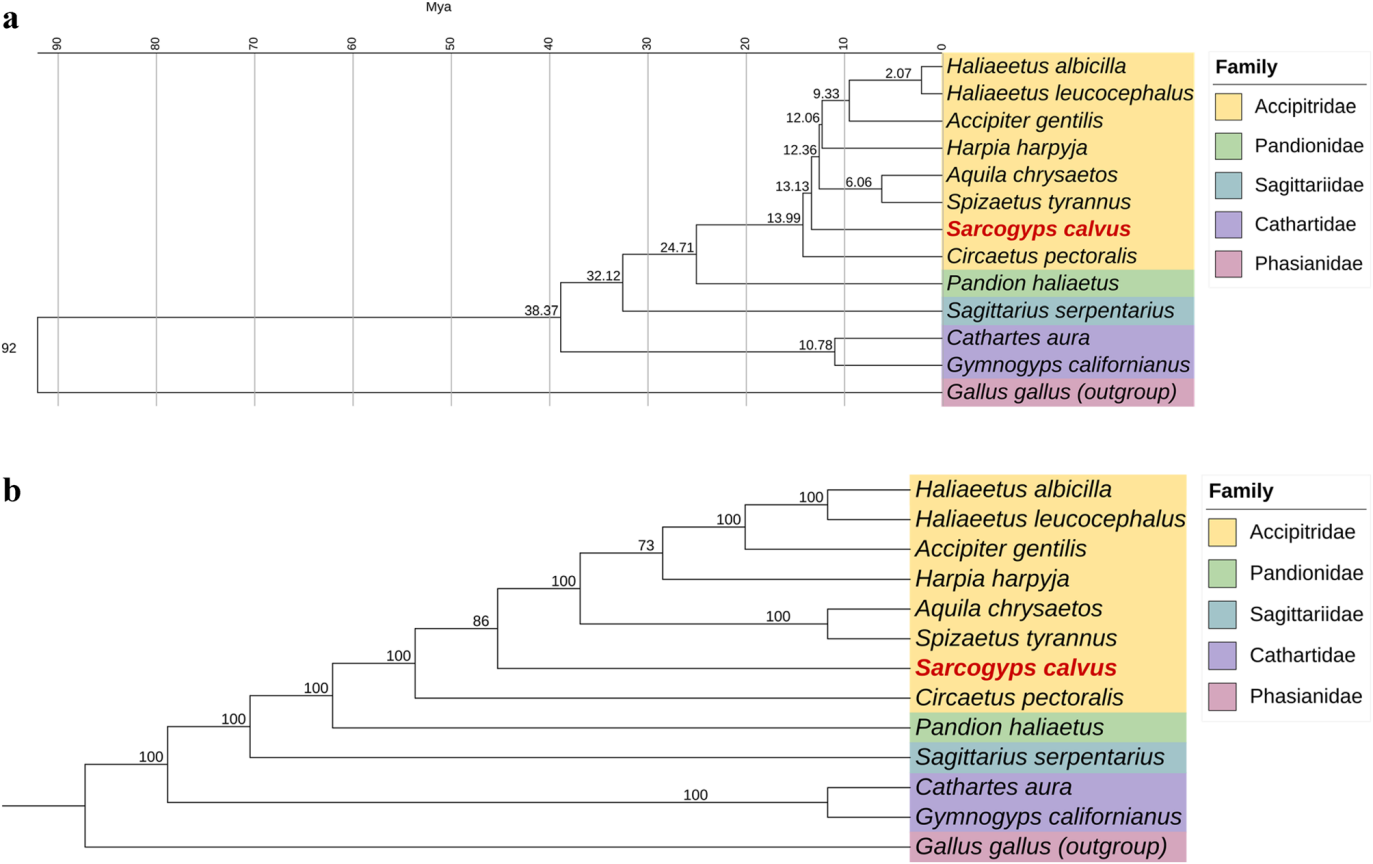
Phylogenetic tree based on amino acid sequences derived from 1259 single-copy orthologous genes of *Sarcogyps calvus* and 11 avian species in the Order Accipitriformes, with red junglefowl (*Gallus gallus*) serving as the outgroup. (**a)** Chronogram. Estimated divergence times are indicated at each node. (**b**) Cladogram. The number at each node represents bootstrap values of 1000 replicates. *Sarcogyps calvus* is highlighted in red.

To illustrate the genomic relatedness among species, a phylogenetic tree based on the nuclear genome was constructed using amino acid sequences of 1259 single-copy orthologous genes from 13 species. These species included 10 birds in the order Accipitriformes: white-tailed eagle (*Haliaeetus albicilla*)*,* bald eagle (*Haliaeetus leucocephalus*), northern goshawk (*Accipiter gentilis*)*,* harpy eagle (*Harpia harpyja*), black hawk-eagle (*Spizaetus tyrannus*), golden eagle (*Aquila chrysaetos*), Asian king vulture (*S. calvus*), black-chested snake eagle (*Circaetus pectoralis*), osprey (*Pandion haliaetus*) and secretary bird (*Sagittarius serpentarius*). Additionally, two NWV species, California condor (*Gymnogyps californianus*) and turkey vulture (*Cathartes aura*), were also included, with red junglefowl (*Gallus gallus*) serving as the outgroup (Fig. 2). In addition, the estimated divergence time between AKV and other Accipitrid hawks and eagles within the same family was approximately 13.13 million years ago (Mya), while the estimated divergence time between AKV and the NWV was approximately 38.37 Mya.

### Species-specific orthologous gene families

**Figure 3:**
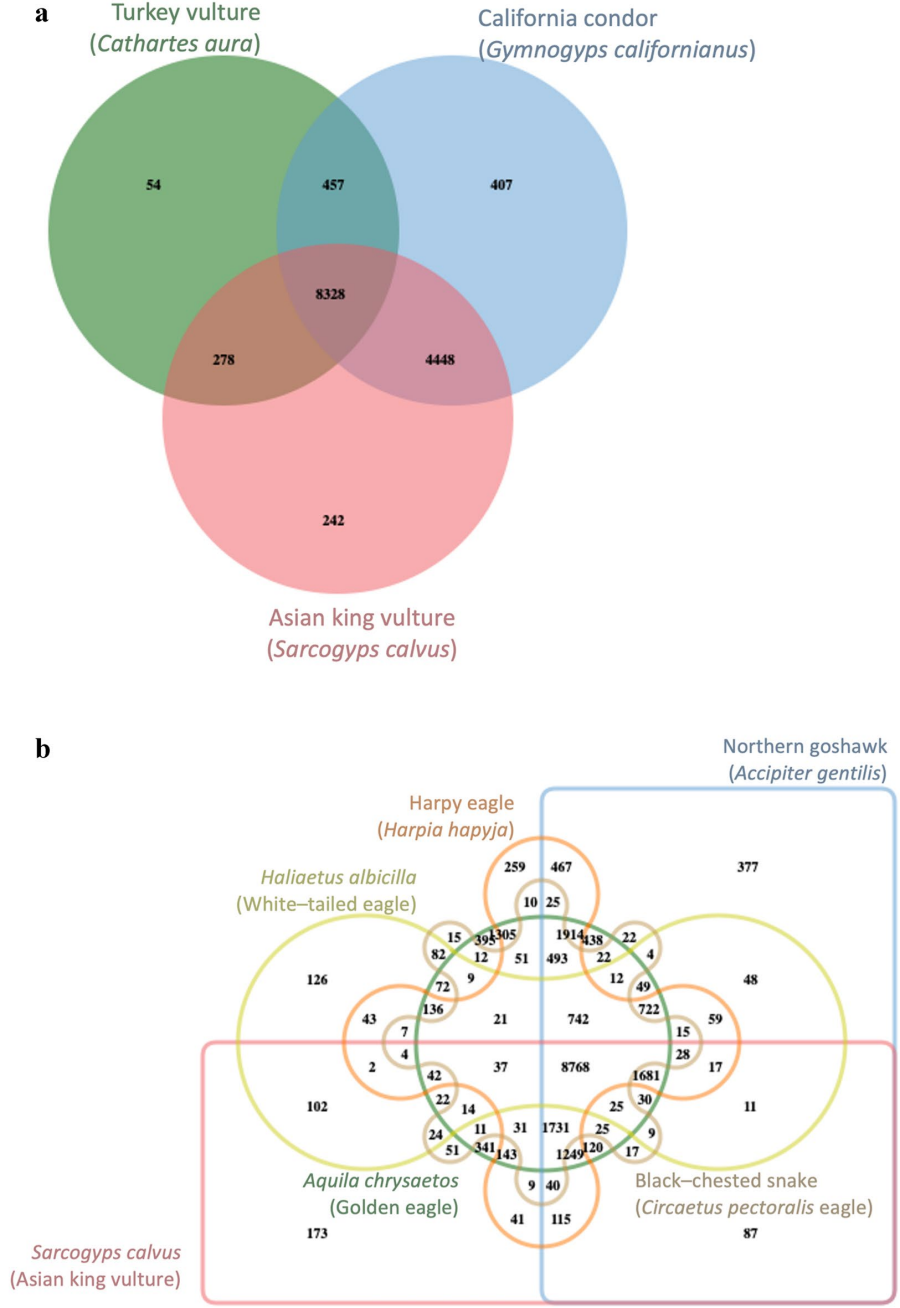
Venn diagrams of species-specific gene families. (**a**) Vultures. (**b**) Accipitridae clade.

Species-specific gene families in the genomes of three vulture species, the AKV, California condor and turkey vulture, were analyzed using OrthoVenn2^21^. Among a total of 14,214 families, 5183 were orthologous families being shared between two species, while 703 gene families were unique to a single species. Among the 242 AKV-specific gene families, approximately 65% (158 families) could be annotated using the UniProtKB/Swiss-Prot database. GO enrichment analysis revealed major families for Gag-Pol polyprotein, associated with viral genome integration to host DNA (GO:0044826), families for envelope glycoprotein, involved in virion attachment to the host cell (GO:0019062), and families for E3 ubiquitin-protein ligase ICP0, associated with the suppression of host interferon regulatory factor 7 (IRF7) activity (GO:0039557) (Fig. 3a, Supplementary Table 4). For the NWVs, 457 gene families were specific to this group. Out of 430 annotated gene families, 358 were associated with biological processes, 46 with molecular functions and 26 with cellular components. The primarily enriched specific gene families of NWVs included families for olfactory receptor families, associated with smell perception (GO:0007608, GO:0004984) (Fig. 3a, Supplementary Table 4).

Notably, species-specific genes in the AKV genome were also compared within the Accipitridae clade, which includes the white-tailed eagle, northern goshawk, harpy eagle, golden eagle and black-chested snake eagle. Out of 22,952 families, 20,001 orthologous families were found in at least two species with 2951 observed as single-copy gene families. A total of 173 AKV-specific gene families were represented in a Venn diagram (Fig. 3b). Among these, 78 gene families could be annotated using the SWISS-PROT database. Similar to the results for species-specific gene families among the three vulture species, we observed gene families for envelope glycoprotein, which is related to virion attachment to host cell (GO:0019062). Additionally, we found other families corresponding to retrovirus integration including families for Gag-Pol polyprotein associated with virion assembly (GO:0019068), families for E3 ubiquitin-protein ligase IE61 involved in the modulation of host protein ubiquitination (GO:0039648), families for Pro-Pol polyprotein associated with viral penetration into the host nucleus (GO:0075732), families for Gag-Pol polyprotein associated with DNA recombination (GO:0006310), and families for Gag polyprotein associated with viral budding via the host ESCRT complex (GO:0039702) (Supplementary Table 5).

### Expansion and contraction of gene families

**Figure 4:**
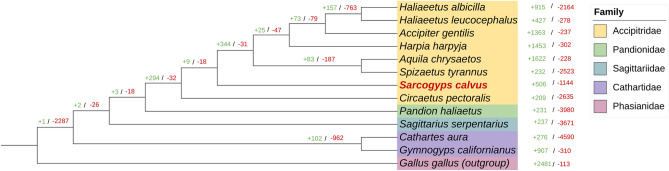
Phylogenetic tree showing gene family evolution. The number of expanded (green) and contracted (red) gene families is displayed at the nodes. The changes in gene family size were analyzed using CAFE software (v5) and visualized using the iTOL.

Gene family expansion and contraction analysis using CAFE v5^22^ revealed 509/1144, 907/310 and 276/4590 gene families expanded/contracted in AKV (*S. calvus*), California condor (*G. californianus*) and turkey vulture (*C. aura*), respectively (Fig. 4). Within these gene families, we observed 31 expanded and 13 contracted gene families that were significantly associated with the AKV. Notably, the major biological process for expansion was acute-phase response (GO:0006953), involving the families for differentiation 163 (CD163) and hemochromatosis (HFE), displaying a fold enrichment exceeding 60. Additionally, the regulation of cellular protein localization (GO:1903827) featured the families for adenylate cyclase 10 (ADCY10), centrosomal protein 250 (CEP250) and HFE with a fold enrichment of 7.66. Moreover, the multi-organism reproductive process (GO:0044703) involved the families for membrane anchored junction protein (MAJIN), sperm associated antigen 4 (SPAG4), ADCY10 with HFE with a fold enrichment of 5.48 (Supplementary Table 6).

### Positively selected genes

Thirteen avian genomes were analyzed for PSGs, including 12 from Accipitriformes and one from Galliformes. The results revealed the presence of 47 significant PSGs in AKV. Several genes with single amino acid substitutions were identified, notably, baculoviral IAP repeat containing 5 (*BIRC5*), *CD274*, aryl hydrocarbon receptor (*AHR*), aggrecan (*ACAN*), myoferlin (*MYOF*), actinin alpha 4 (*ACTN4*), mannose receptor C-type 1 (*MRC1*) and spermidine synthase (*SRM*). Predictions suggest that these substitutions may negatively impact the genome when compared to the red junglefowl reference sequence. The PSG families associated with these genes revealed significant enrichment in various biological processes. Notably, the positive regulation of the cell cycle checkpoint (GO:1901978) showed the highest fold enrichment, with the *BIRC5* gene displaying a fold enrichment exceeding 50. Additionally, the response to tumor cells (GO:0002834) featured *CD274* and *AHR* genes with a fold enrichment surpassing 40. The third-ranked process implicated the regulation of stem cell proliferation (GO:0072091), with *ACAN* exhibiting a fold enrichment of 10.53, while muscle cell development (GO:0055001), containing *MYOF* and *ACTN4*, demonstrated a fold enrichment of 9.73. Furthermore, cellular response to cytokine stimulus (GO:0071345) was observed, involving *CD274*, *MRC1*, *ACTN4* and *SRM* genes, with a fold enrichment of approximately 3. The major molecular functions were actin binding (GO:0003779), associated with the *ACTN4* gene, and protein dimerization activity, which encompassed the *ACTN4*, *BIRC5*, *AHR* and *SRM* genes. (Supplementary Tables 7, 8).

### SNP and genotype calling

RADseq was performed on whole blood samples from five AKVs. The raw reads were aligned to our de novo AKV assembly, resulting in 626,584 SNPs. SNPs on Z and W chromosomes were filtered out after aligning against the Z and W chromosomes of the golden eagle (Accession No GCF_900496995.4) to remove low quality SNPs and eliminate potential bias from SNPs on putative sex chromosomes. After filtering, 52,167 SNPs remained. Subsequently, two SNP datasets were prepared, including a non-linkage disequilibrium (LD)-pruned dataset and an LD-pruned dataset. The non-pruned LD dataset, containing 52,167 SNPs, was used for detecting runs of homozygosity (ROH) to avoid underestimation^23^. The pruned LD dataset, resulting in 2,220 SNPs, was utilized for phylogenetic relationship, multidimensional scaling (MDS) and pairwise identical by descent (IBD) relationship analyses.

### Population structure and genetic relatedness based on identity by descent

**Figure 5:**
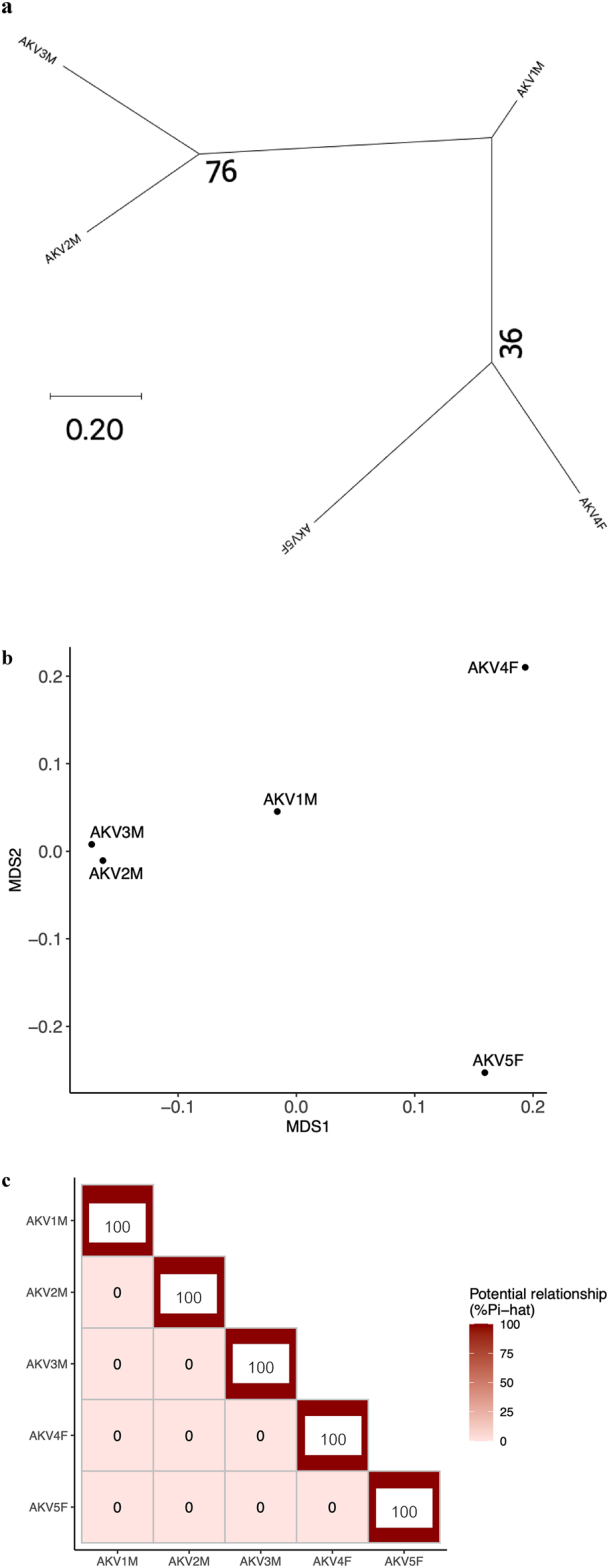
Population genomics of five Asian king vultures in Thailand. (**a**) Phylogenetic tree of captive AKV population. (**b**) Multidimensional scaling (MDS) plot of AKV population. (**c**) Heatmap of Pi-hat coefficient of AKV pairs. F, female; M, male.

In the context of phylogenetic analysis, AKV2M and AKV3M were found to form a sister group, as did AKV4F and AKV5F. AKV1M, AKV2M and AKV3M exhibited a closer genetic similarity with shorter branch lengths when compared to AKV4F and AKV5F, implying that AKV1M, AKV2M and AKV3M likely belong to the same subpopulation (Fig. 5a). The MDS plot illustrated three potential clusters for those five AKV samples. The initial cluster comprised AKV1M, AKV2M and AKV3M; the second cluster consisted of AKV4F, while the third cluster encompassed AKV5F (Fig. 5b). Additionally, the IBD analysis, as depicted in the heatmap (Fig. 5c), indicated that all five AKV individuals were genetically unrelated.

### Inbreeding evaluation

**Figure 6:**
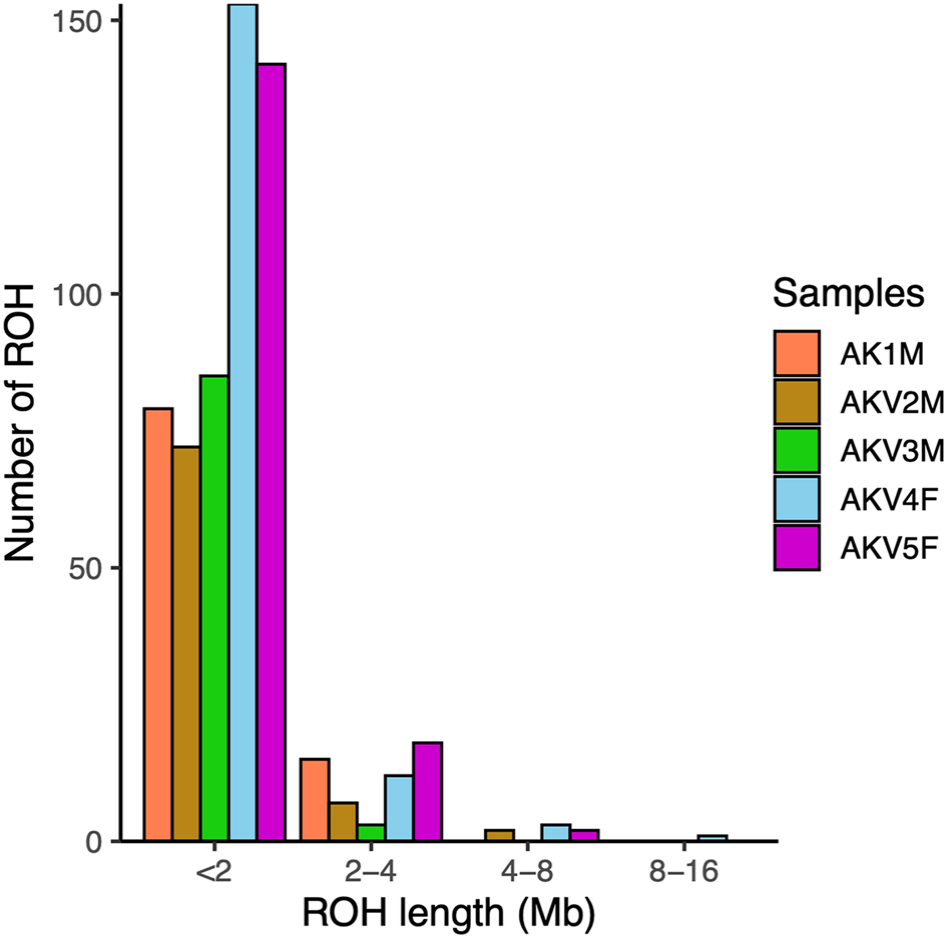
Runs of homozygosity (ROH) analysis of Asian king vulture population, calculated using PLINK v1.9 and the detectRUNS package.

The inbreeding coefficients computed from the ROH length (F_ROH_) for the five AKV individuals were as follows: AKV1M = 0.09, AKV2M = 0.08, AKV3M = 0.07, AKV4F = 0.17 and AKV5F = 0.15. Their average F_ROH_ was 0.11 ± 0.05. In addition, we calculated the inbreeding coefficient based on excess homozygosity (F_HOM_) using PLINK. The genetic diversity indices for each individual AKV, including observed and expected homozygosity and F_HOM_, are summarized in Table 2. We observed a positive correlation between F_ROH_ and F_HOM_ (Pearson’s correlation test, correlation coefficient = 0.98, *p* < 0.01). The relationship between the ROH count and the ROH length is illustrated in Fig. 6. Notably, AKV4F and AKV5F exhibited the longest ROH lengths, exceeding 16 Mb.

**Table 2:** Statistics of genetic diversity indices of AKV.

Samples	Observed number of homozygotes	Expected number of homozygotes	No. of non–missing genotypes	F_HOM_
AKV1M	1214	1154	2220	0.05584
AKV2M	1232	1154	2220	0.07274
AKV3M	1217	1154	2220	0.05866
AKV4F	1340	1154	2220	0.1741
AKV5F	1336	1154	2220	0.1703
mean ± SD	1267.8 ± 64.46	1154 ± 0.00	2220 ± 0.00	0.11 ± 0.06

F_HOM_, inbreeding coefficient from excess homozygosity.

## Discussion

This study presented the first reference genome of AKV and conducted comparative genomic analyses with published genomes of NWVs, including California condor and turkey vulture, as well as other bird species within the Accipitridae family. Additionally, the RADseq-based genome-wide SNPs were utilized to elucidate the genetic relatedness among five AKVs in Thailand. The AKV reference genome assembly was constructed from raw reads obtained through a combined PacBio-MGI-Purged method. Purge_dups analysis was employed to identify and eliminate duplicate regions, haplotigs and heterozygous overlaps, resulting in enhanced assembly continuity while preserving completeness. Although the MinION portable sequencer theoretically provides up to 20 Gb of data^24^, in the present study, 13.5 Gb of raw reads were obtained from two flow cells, generously supported by ORG.one. Given the AKV genome size of 1.29 Gb, at least six flow cells would be required to achieve 30 × coverage. Consequently, the output was insufficient for a whole-genome assembly of the AKV. Nonetheless, the AKV genome assembly achieved a scaffold N50 of 28.73 Mb and a genome size of 1.29 Gb, surpassing those of the NWVs. Specifically, the California condor has a scaffold N50 value of 17.1 Mb and a genome size of 1.24 Gb, whereas the turkey vulture has a scaffold N50 value of 15.2 Kb and a genome size of 1.15 Gb^25,26^. Moreover, when compared to closely related taxa with scaffold N50 values ranging from 0.057 to 58.1 Mb and genome sizes ranging from 1.18 to 1.27 Gb, the AKV genome size falls within this range of scaffold N50 lengths. The completeness of the AKV genome, at 97.4%, is comparable to that of sister taxa (white-tailed eagle*,* bald eagle, northern goshawk*,* harpy eagle, black-hawk eagle and golden eagle), which exhibit genome completeness ranging from 91.2 to 99.2%^26–28^. It is noteworthy that different sequencing techniques may impact results, as most of the published reference genomes mentioned above were generated using Illumina short-read sequencing, except for the genomes of the California condor, harpy eagle, golden eagle and northern goshawk, which were sequenced using both Illumina short-read and PacBio long-read sequencing. Additionally, genomes with larger scaffold N50 values were assembled at the chromosome level^27,28^. The AKV genome assembly revealed that repetitive elements constitute 7.8% of the genome. Repetitive elements in avian genomes typically range from 4 to 10% of the genome assembly, except for the downy woodpecker, which contains repetitive elements accounting for 22% of the genome assembly due to species-specific LINE expansions^29^. Using a combination of short-read and long-read sequencing, along with advanced bioinformatic tools, could potentially enhance the annotation of repetitive elements^30^.

In this study, a total of 19,388 predicted protein-coding genes were identified in the AKV. When compared to other species available in the NCBI database, the turkey vulture (accession number: GCA_000699945.1) and the California condor (accession number: GCA_018139145.2) were found to have 11,361 and 15,144 protein-coding genes, respectively. Conversely, species more closely related to AKV, such as the golden eagle (accession number: GCA_900496995.4) and bald eagle (accession number: GCA_000737465.1), were annotated with 16,702 and 15,212 protein-coding genes, respectively. It is worth noting that gene annotation using RNA sequencing-based methods likely contributed to improved annotations^31^. Additionally, divergence time estimation was conducted based on 1259 single-copy orthologous genes between AKV and other species. The estimated divergence time between OWVs and NWVs was determined to be 38.37 Mya, while the estimated divergence time between AKV and sister taxa, including the white-tailed eagle*,* bald eagle, northern goshawk*,* harpy eagle, black hawk-eagle and golden eagle, was estimated to be 13.13 Mya. These estimations differ from previous studies, where the estimated divergence time between OWVs and NWVs was 60 Mya, and between cinereous vultures and bald eagles, it was 18 Mya^15^. These variations may arise from differences in the methods and calibration points utilized in the analysis.

In our comparative genomic analysis, the investigation of species-specific gene families revealed enriched gene families associated with virus infection. These functions included virion attachment to host cells, viral genome integration, virion assembly and budding. Endogenous retroviral fragments were identified in several bird species^32^. Among vultures, retroviral genome integration was previously documented solely in the turkey vulture genome, while avian endogenous retrovirus EAV-HP was reported in the facial skin microbiome of both black vultures and the turkey vultures^32,33^. Notably, this study marks the first identification of the retrovirus-related sequences in the AKV genome. Furthermore, our analysis revealed the E3 ubiquitin-protein ligase ICP0 families, associated with the suppression of host defense responses. Within the ubiquitin proteasome system, E3 ubiquitin ligases play a crucial role in transferring ubiquitin to target substrates for subsequent proteolysis^34^. Specifically, ICP0 mediates the degradation of IRF7, a regulator of type I interferon production, which is a crucial cytokine for both innate and adaptive immunity^35^.

In terms of gene families or genes that were associated with the immune response and host defense mechanisms, certain highly enriched expanded gene families, such as CD163 and HFE, have been shown to be associated with immune responses and iron regulation. CD163 facilitates the endocytosis and clearance of hemoglobin/haptoglobin complexes by macrophages, potentially protecting tissues from oxidative damage caused by free hemoglobin and serving as an innate immune sensor for microorganisms^36^. Additionally, HFE plays a significant role in the inflammatory response by modulating CD8^+^ T-lymphocyte responses due to its structural resemblance to MHC I^37^. In addition, HFE is implicated in iron regulation by inhibiting iron efflux from macrophages and enterocytes, potentially influencing susceptibility to bacterial infections due to iron’s role as a nutrient for various pathogens^38^.

Apart from the expanded gene families, several PSGs exhibited substantial enrichment in pathways associated with the immune response and defense mechanisms. Notable examples include *CD274*, *AHR*, *CYP1A2*, *MRC1*, *BIRC5* and *SRM* genes. *CD274* is identified as an adhesion molecule present on T-cells, antigen-presenting cells and tumor cells according to KEGG pathways (map04514 and map05235). It plays an important role in the PD1/PD-L signaling pathway, regulating inappropriate immune responses^39^. AHR has been reported to activate Th17 differentiation in response to inflammatory stimuli^40^. Additionally, AHR is involved in xenobiotic metabolism by mediating *CYP1A* expression^41^. *CYP1A* in turn, plays pivotal roles in biotransformation of exogenous substances such as drugs and pollutants, as well as endogenous substances like hormones, vitamins and fatty acids^42^. MRC1, a glycoprotein located on the surface of antigen presenting cells, is crucial for cell–cell and pathogen recognition, mediating T-cell activation, antigen processing and presentation^43^. *BIRC5*, involved in positive regulation of the cell cycle response, is highly expressed in T cells and various tumor tissues^44,45^. Its high expression in tumors correlates positively with immune cell activation, suggesting its role as an immune-related gene^46^. Finally, *SRM* plays a pivotal role in spermidine biosynthesis, a natural polyamine with diverse functions including antioxidant properties, regulation of cell division, protein synthesis, tissue growth and reduction of lipid accumulation in adipose tissue^47,48^. However, the role of these gene families and genes in vultures should be further investigated.

In addition to host defense mechanisms, several genes or gene families with distinct yet related functions have been uncovered. These include species-specific gene families for feather keratin, as well as *MYOF* and *ACTN1*, which are highly enriched PSGs associated with muscle cell development. Beta keratin serves as a primary component of feather shafts, crucial for imparting stiffness and strength to withstand significant aerodynamic forces encountered during high-altitude soaring^49^. *MYOF* is expressed in both cardiac and skeletal muscle and plays a crucial role in muscle development by regulating the canonical Wnt signaling pathway^50,51^. Meanwhile, *ACTN1* and *ACTN4* are involved in actin filament bundling, cell adhesion and cell migration in non-muscle cells^52,53^. Apart from the AKV’s gene families, prominent NWV-specific gene families have been linked to the sense of smell, including more than 10 olfactory receptor gene families. This finding aligns with previous studies, indicating that NWVs possess a heightened sense of smell attributed to a greater number of OR families compared to birds in the Accipitridae family^6^.

In terms of genetic relationships within the small population, SNPs located on putative Z and W chromosomes were excluded to mitigate potential bias stemming from sex chromosomes. This exclusion ensures a more accurate evaluation of genetic relatedness across the autosomal genome, allowing for a clearer understanding of population structure and genetic diversity. Despite the fact that these AKVs were donated at different time points and lack available pedigree or records, both phylogenetic tree analysis and MDS plot consistently revealed distinct subpopulations among the five AKV individuals. These subpopulation divisions were determined through genetic distance analyses based on identical by state (IBS) values. Upon the evaluation of inbreeding status, AKV4F and AKV5F exhibited a high proportion of ROH (F_ROH_), indicating the possibility of parental consanguinity or close cousin mating for each animal. It is noteworthy that population history, particularly in species with a low effective population size, possibly influences ROH values and density^54^. From our data, it can be inferred that AKV4F and AVK5F likely originate from bottlenecked subpopulations that shared recent common ancestors. High genome-wide homozygosity rates or high F_ROH_ values are correlated with an increased risk of inbreeding depression, which has previously been associated with reproductive issues such as decreased fledgling production^55,56^.

Despite achieving success in breeding AKVs for the first time in 30 years, Thailand faces challenges as the remaining AKV breeders are aging, with a mean age of approximately 25.4 ± 3.6 years, and they lay eggs approximately once every 2 years. Furthermore, captive OWVs were reported to lay their first eggs at 7–9 years old, with the first offspring reproduced at 8–10 years of age^57^. This suggests that the repopulation of AKVs in Thailand may encounter a founder effect crisis in the next generation. To support the global conservation efforts of AKVs, it is crucial to investigate the genetic relatedness of captive AKVs worldwide, employing methods like affordable RADseq-based genome-wide SNPs, to facilitate breeding programs. Additionally, assisted reproductive technology (ART) techniques such as semen cryopreservation and artificial insemination should be integrated. This combined approach can help mitigate the adverse effects of closed genetic relatedness and contribute to the long-term health and sustainability of the population, setting a valuable model for conservation efforts in other endangered species.

## Conclusion

Our study presents the first comprehensive genomic analysis of the AKV, shedding light on unique genetic features likely associated with their adaptation to scavenging carrion and soaring at high altitudes as well as potential mechanisms for modulating host defenses. Population genomics, utilizing genome-wide SNP data from RADseq, revealed that the AKV individuals were not genetically related to one another. However, two female AKVs showed a high risk of inbreeding. The utilization of genome-wide SNP data shows promise in guiding breeding programs for animals with limited pedigree information and assessing genetic relatedness before introducing individuals from different locations for breeding purposes. These insights significantly contribute to the conservation efforts of the AKV and hold potential relevance for other species facing similar challenges.

## Methods

### Ethical approval

All methods were performed in accordance with guidelines and regulations and the study was carried out in compliance with the ARRIVE guidelines. All animals were managed following the ethical guidelines required under the Chulalongkorn University Animal Care and Use Committee (CU–ACUC), Thailand (approval number 2131005).

### Animals

Whole blood samples were obtained from five AKV individuals. Four of them were from ZPOT, comprising three males (AKV1M, AKV2M, AKV3M) aged 22, 21 and 27 years, respectively, and one female (AKV4F) aged 25 years. The fifth individual, a female (AKV5F) approximately 17 years old, was obtained from Huai Kha Khaeng Wildlife Breeding Center, DNP. All five AKV individuals were donated a long time ago and do not have any recorded pedigree information.

### De novo whole genome sequencing and assembly

Genomic DNA was extracted from a whole blood sample of a 25-year-old female AKV (AKV4F) using a Wizard HMW DNA Extraction Kit (Promega, Madison, WS, USA). Genomic DNA quantity was measured using a NanoDrop One Microvolume UV–Vis Spectrophotometer (Thermo Fisher Scientific, Waltham, MA, USA), and quality and integrity were assessed using pulsed-field gel electrophoresis. We performed hybrid sequencing using the Pacific Biosciences (PacBio) Sequel II platform for long-read sequencing and the MGISEQ-2000 platform for short-read paired-end sequencing. Raw reads from PacBio were assembled using Canu v2.2.0. Pilon was utilized for polishing the draft genome with short-read data. Redundant haplotigs and contig overlaps were removed using purge_dups v1.2.6. The completeness of the assembled genome was analyzed by searching against single-copy orthologs (avian gene set; *n* = 8,338) using BUSCO v4.0.5.

### Genome annotation and prediction

RepeatModeler version 2.0.2 (http://www.repeatmasker.org/RepeatModeler) was utilized to generate a de novo repeat library and identify transposable element (TE) families within the unannotated genome assembly. Additionally, RECON version 1.08 and RepeatScout version 1.0.5 were employed to delineate the boundaries of repetitive elements and construct consensus models for interspersed repeats. To ensure that repeat sequences in the library did not contain large families of protein-coding genes that are not transposable elements, an alignment to GenBank’s non-redundant protein database was conducted using BLASTX with an *E*-value cutoff of 1 × 10^–6^. Repeat masking was carried out on the assembled genome using RepeatMasker version 4.0.6 (http://www.repeatmasker.org/) against the repetitive sequences in the RepeatMasker consensus library (20,150,807; www.girinst.org).

Gene models for the repeat-masked genome were annotated using a combination of homology-based prediction, RNA-based prediction and ab initio prediction techniques. This process facilitated the identification of protein-coding sequences, using the MAKER2 software. Regarding RNA evidence, transcript sequences from *H. leucocephalus* were aligned to the assembled sequences using the exonerate tool. For homology prediction, protein sequences from four avian species (*G. gallus*, *Meleagris gallopavo*, *Taeniopygia guttata* and *Columba livia*) were retrieved from the UniProtKB database (www.uniprot.org) and aligned to the genome using the exonerate tool within the MAKER annotation pipeline. Ab initio predictions were generated using Augustus, version 3.3.3 (Augustus: Gene Prediction, RRID:SCR_008417), with parameters derived from training with red junglefowl genes. Additionally, SNAP was utilized for another set of ab initio predictions, with parameters computationally optimized through a training process during the first round of the MAKER annotation pipeline.

To identify and classify de novo repeat families, RepeatModeler version 1.0.11 (http://www.repeatmasker.org/RepeatModeler) was run on the unannotated assembly. Putative short noncoding RNAs (ncRNA) were identified using structRNAfinder, which integrates different tools (Infernal and RNAfold) to facilitate ncRNA annotation based on sequence/covariance model comparisons and databases, including the Rfam database (release 12.0) for cross-referencing. Furthermore, tRNAs were annotated using tRNAscan-SE, version 1.3.1, with default parameters. Functional annotation of the genome relied on annotations from the Nr, SwissProt, GO and KEGG databases.

### Phylogenetic analysis

Single-copy orthologous protein sequences from 14 species, including *S. calvus, A. gentilis, A. chrysaetos, C. pectoralis, H. albicilla, H. leucocephalus*, *H. harpyja*, *S. tyrannus, C. aura, P. haliaetus, S. serpentarius, G. gallus* and *H. sapiens*, were utilized for the analysis. Initially, these protein sequences underwent multiple sequence alignment using prank v170427^58^ and were subsequently employed to construct a phylogenetic tree with IQ-TREE v2.2.0.3^59^. The model for tree reconstruction was selected using ModelFinder within the same program^60^. The phylogenetic tree was generated with 1000 ultrafast bootstrap replicates^61^. The divergence time of AKV (presumably referring to one of the species) was estimated using r8s v1.81^62^. The calibration points between *G. gallus* and *H. leucocephalus*, obtained from www.timetree.org, was utilized as input for estimating the divergence time.

### Species-specific gene families, gene family expansion and contraction

OrthoVenn2 was conducted using protein sequences from each species as input to identify species-specific gene families^21^. Specifically, the gene families between the AKV (OWV) and the turkey vulture and California condor (NWVs) were compared. Additionally, comparisons between AKV and other birds in the Accipitridae family were conducted, which encompassed species such as *A. gentilis*, *A. chrysaetos*, *H. albicilla*, *H. harpyja* and *C. pectoralis*. To analyze gene family expansion and contraction, we employed CAFE v5^22^ and the gene birth-date parameters were calculated to estimate the time when gene duplication events occurred using the maximum-likelihood method. Rapidly expanding and contracting gene families were identified at a significance level of *p* < 0.05. GO enrichment analysis of significantly expanded gene families was performed using the DAVID database.

### Positively selected genes

Coding sequences from 12 species in Accipitriformes and one species in Galliformes were utilized to analyze genes under positive selection using the branch-site model in CODEML implemented in PosiGene^63,64^. PSGs were identified based on criteria such as dN/dS > 1, *P*-value < 0.05 and FDR-adjusted *P*-value (Q-value) < 0.05. The significance of positive selection on these genes and the fold enrichment of specific biological processes and molecular functions associated with PSGs were analyzed using the DAVID database. In addition, the potential effects of amino acid replacements on protein functions were predicted using the Protein Variation Effect Analyzer (PROVEAN) software, compared with the red junglefowl genes^65^. The red junglefowl genome was selected as a reference because all PSGs of AKV were observed in the red junglefowl genome. GO enrichment analysis was also performed using the DAVID database.

### Restriction-site associated DNA sequencing (RADseq)

Five AKV whole blood samples, comprising three males (AKV1M, AKV2M and AKV3M) and two females (AKV4F and AKV5F), were subjected to for genomic DNA extraction utilizing the Quick-DNA Miniprep Plus kit (Zymo Research, Irvine, CA, USA) according to the manufacturer’s protocol. DNA concentrations were determined using both a NanoDrop One Microvolume UV–Vis Spectrophotometer (Thermo Fisher Scientific) and a Qubit fluorometer (Life Technologies). Assessment of sample integrity was conducted via pulsed-field gel electrophoresis. For library preparation, the DNA samples were diluted to a final concentration of 0.05 g/L. The RAD library was constructed following the manufacturer’s instructions, employing the MGIEasy RAD Library Prep Kit (MGI Tech, Shenzhen, China). To achieve this, 1 µg of genomic DNA underwent enzymatic fragmentation using two restriction enzymes, *Taq*I and *Mse*I. Subsequently, RAD adapters and barcodes were employed to ligate digested fragments. All resultant products from the various samples were pooled, and the quality of library construction was assessed using the Fragment Analyzer System (Agilent Technologies, Santa Clara, CA, USA). The final PCR-amplified library fragments, ranging within the 400 − 450 bp, were transformed into a single-strand circularization DNA library and sequenced using the MGI Tech MGISEQ-2000RS platform, generating 150-bp paired-end reads. Raw reads were demultiplexed based on their barcodes, followed by the removal of sequences containing adaptors, barcodes, and low-quality reads from the dataset.

### SNP calling and filtering

Raw reads obtained from RADseq were aligned to the reference genome, assembled in this study, utilizing Bowtie2 v2.3.5.1^66^. The resulting SAM files underwent conversion to BAM files using Samtools v1.10^67^. All BAM files were then utilized for SNP calling employing GATK v4.4.0.0. SNPs located on Z and W chromosomes were aligned against the Z and W chromosomes of the golden eagle (Accession No. GCF_900496995.4) using minimap2^68^. These SNPs were filtered out using vcftools (with parameters—remove-indels, —max-missing 1, minQ 30, minDP 3 and —not-chr for contigs likely containing sex-related sequences) and PLINK v1.9 (applying —maf 0.05, —geno 0, —mind 0 and —hwe 0.01) to eliminate low quality SNPs and mitigate potential biases arising from SNPs on putative sex chromosomes. Two SNP datasets were prepared: a non-LD-pruned dataset and an LD-pruned dataset. The latter was generated utilizing the parameters ––indep-pairwise 50 5 0.2, specifying a 50-SNPs sliding window, a 5-SNP step size for shifting the window and an r^2^ threshold of 0.2 for pruning.

### Population structure and genetic relatedness

For the analysis of population structure, the LD-pruned dataset underwent analysis through phylogenetic tree construction and MDS plot generation. A phylogenetic tree was constructed using MEGA X software, employing the maximum likelihood method. The General Time Reversible model was determined to be the best fit for the analysis. The tree construction incorporated 1000 bootstraps to assess the robustness of the tree topology. MDS analysis was conducted using the —mds-plot command in PLINK v1.9.

For the analysis of IBD relatedness, the LD-pruned dataset was utilized to analyze pairwise IBD sharing, based on Pi-hat values. Additionally, probabilities of sharing zero (Z0), one (Z1) and two (Z2) IBD alleles were calculated using the ––genome command in PLINK v1.9. The potential relatedness of each AKV was illustrated in a heatmap using R v4.1.1. For the inbreeding analysis, as there were insufficient LD-pruned SNPs for ROH analysis, the non-LD-pruned dataset was employed to detect ROH in each AKV individual. Sliding-window-based run detection was performed using the detectRUNs package version 0.9.6 within R v4.1.1^69^. Parameters were configured as follows: windowSize = 15, threshold = 0.05, minSNP = 20, maxOppWindow = 1, maxMissWindow = 1, maxGap = 10^6^, minLengthBps = 250,000, minDensity = 1/10^3^, maxOppRun = NULL and maxMissRun = NULL. The ROH segments were categorized by length into the following groups: 0–2 Mb, 2–4 Mb, 4–8 Mb, 8–16 Mb and > 16 Mb. Genome-wide F_ROH_ values were estimated for each AKV. Individuals’ F_HOM_ were identified by analyzing observed homozygosity, expected homozygosity and non-missing genotypes using the ––het command in PLINK. Additionally, a comparison between F_ROH_ and F_HOM_ was conducted by assessing the correlation between them using Pearson’s correlation test.

### Supplementary Information

Supplementary Information.

## Data Availability

The whole genome sequencing data and genome assemblies generated in this study have been deposited in the NCBI database under BioProject PRJNA827941. All other relevant data are available upon request to the corresponding author.
