# Supplementary material for: Comprehensive genome assembly reveals genetic diversity and carcass consumption insights in critically endangered Asian king vultures
Source: Sci Rep. 2024 Apr 24;14:9455. doi: 10.1038/s41598-024-59990-9 (PMC11043450; doi:10.1038/s41598-024-59990-9)
Supplement: Supplementary file 1 — Supplementary Information. [file 41598_2024_59990_MOESM1_ESM.docx]

**Supplementary Table 1.** Repeat elements in the Asian king vulture genome.

| **Repeat element** | **Number of loci** | **Length occupied (bp)** | **Percentage of**  **the assembly** |
| --- | --- | --- | --- |
| Retrotransposons: | | | |
| LINE | 82,642 | 35,396,525 | 2.75 |
| SINE | 4,517 | 491,122 | 0.04 |
| LTR | 73,516 | 38,886,593 | 3.02 |
| DNA elements | 23,675 | 2,846,904 | 0.22 |
| Simple repeats | 290,909 | 11,331,139 | 0.88 |
| Low complexity | 51,527 | 2,540,671 | 0.20 |
| Unclassified | 29,046 | 8,859,145 | 0.69 |

**Supplementary Table 2.** Statistics on non-coding RNA (ncRNA) in the Asian king vulture genome.

| **Type** | **Number** | **Total length (bp)** | **Mean length (bp)** |
| --- | --- | --- | --- |
| rRNA | 243 | 148,875 | 612.65 |
| microRNA | 6,424 | 521,183 | 81.13 |
| snRNA | 14,389 | 997,268 | 69.31 |
| snoRNA | 13,907 | 946,765 | 68.08 |
| splicing | 482 | 50,503 | 104.78 |
| tRNA | 515 | 38,581 | 74.91 |
| other ncRNA | 9,853 | 972,098 | 98.66 |

**Supplementary Table 3.** Characterized endogenous retroviral (ERV) genes in the Asian king vulture genome.

| **ERV genes** | **Group** | **Copy number** | **%Total ERV sequence** |
| --- | --- | --- | --- |
| *env* | 3 | 46 | 23.71 |
| *env* | V | 18 | 9.28 |
| Group-specific antigen (*gag*) | K | 31 | 15.98 |
| *pol* | K | 99 | 51.03 |

**Supplementary Table 4.** Functional enrichment of Asian king vulture (AKV)-specific families compared to two New World vultures (turkey vulture and California condor).

| **Term** | **Category** | **Description** | **Number of family** | ***P*-value** | **Swiss-Prot ID** | **Protein** |
| --- | --- | --- | --- | --- | --- | --- |
| **Functional enrichment of AKV-specific families compared to two New World vultures:** | | | | | | |
| GO:0044826 | Biological process | viral genome integration into host DNA | 8 | 1.42E-14 | Q7SQ98, P03360, P31623, P11283 and P31792 | Gag-Pol polyprotein |
| GO:0019062 | Biological process | virion attachment to host cell | 4 | 1.26E-06 | P21415, P03385 and P03396 | Envelope glycoprotein, Envelope glycoprotein gp95 |
| GO:0039557 | Biological process | suppression by virus of host IRF7 activity | 2 | 1.39E-04 | P29128 | E3 ubiquitin-protein ligase ICP0 |
| GO:0060074 | Biological process | synapse maturation | 3 | 1.80E-04 | Q8BI27 | Syntabulin |
| GO:0010468 | Biological process | regulation of gene expression | 3 | 1.52E-03 | Q9QYP0 and Q7Z7M0 | Multiple epidermal growth factor-like domains protein 8 |
| GO:0005200 | Molecular function | structural constituent of cytoskeleton | 7 | 2.65E-08 | Q92012, P02449, P08335 and Q9PSV3 | Beta-keratin-related protein, Feather keratin, Feather keratin B-4 and  Feather keratin Cos2-3 |
| **Functional enrichment of New World vulture-specific families compared to Asian king vulture:** | | | | | | |
| GO:0007608 | Biological process | sensory perception of smell | 11 | 2.67E-10 | Q8VGR8, Q8VFK7, Q8NGX0, Q60894, Q8VGS3, A6ND48, Q8NHC5, Q8N0Y5, Q8N127 and P0C626 | Or5j3, Or5ap2, OR11L1, Or9s13, Or5ar1, Or5j3, OR14I1, OR14A16, OR8I2, OR5AS1 and OR5G3 |
| GO:0004984 | Molecular function | olfactory receptor activity | 10 | 8.00E-09 | Q9UGF6, Q96KK4, Q8NGE5, Q8NH08, O95007, Q8NGX8, Q8NGZ4, Q9UGF6, O95007 and P0C7N8 | OR5V1, OR10C1, OR10A7, OR10AC1, OR6B1, OR6Y1, OR2G3, OR5V1, OR6B1 and OR9G9 |
| GO:0001835 | Biological process | blastocyst hatching | 3 | 1.28E-04 | Q8C811 and Q2KIK3 | Slc35e2 and SMIM14 |
| GO:0008270 | Molecular function | zinc ion binding | 8 | 6.46E-04 | Q9H8U3, Q9BY12, A6QQX9, Q9HCJ6, Q96AW0, A1L2T6, Q9P2E8 and B2RVL6 | ZFAND3, SCAPER, TMEM163, VAT1L, DTNB, zcchc7.L, MARCHF4 and Zcchc24 |

# Supplementary Table 5. Functional enrichment analysis of AKV-specific gene families among the Accipitridae clade.

| **Term** | **Description** | **Number of family** | ***P*-value** | **Swiss-Prot ID** | **Protein** |
| --- | --- | --- | --- | --- | --- |
| **Biological process:** | | | | | |
| GO:0019062 | virion attachment to host cell | 8 | 4.73E-15 | P11268, P21415, P03385, P26804 and P03397 | Envelope glycoprotein and envelope glycoprotein gp95 |
| GO:0006313 | transposition, DNA-mediated | 6 | 1.91E-12 | Q95SX7 and Q9NBX4 | Probable RNA-directed DNA polymerase from transposon BS and probable RNA-directed DNA polymerase from transposon X-element |
| GO:0019068 | virion assembly | 6 | 4.54E-10 | Q2F7J3, P10272, Q7SVK7 and P03359 | Gag-Pol polyprotein and Gag-Pol |
| GO:0039648 | modulation by virus of host protein ubiquitination | 3 | 4.87E-08 | P09309 and Q9E1W2 | E3 ubiquitin-protein ligase IE61 |
| GO:0075732 | viral penetration into host nucleus | 3 | 1.94E-07 | P23074 and O93209 | Pro-Pol polyprotein |
| GO:0006310 | DNA recombination | 3 | 4.01E-06 | P63133 and O92815 | Endogenous retrovirus group K member 8 Pol protein and Gag-Pol polyprotein |
| GO:0060074 | synapse maturation | 3 | 5.70E-06 | Q8BI27 | Syntabulin |
| GO:0039557 | suppression by virus of host IRF7 activity | 2 | 4.04E-05 | P29128 | E3 ubiquitin-protein ligase ICP0 |
| GO:0060348 | bone development | 2 | 2.00E-04 | Q8WY36 | HMG box transcription factor BBX |
| GO:0039702 | viral budding via host ESCRT complex | 2 | 5.91E-04 | P21416 and P51516 | Gag polyprotein |
| GO:0010468 | regulation of gene expression | 2 | 3.45E-03 | Q9QYP0 | Multiple epidermal growth factor-like domains protein 8 |
| GO:0007165 | signal transduction | 6 | 3.63E-03 | Q9BZL4 and Q8N103 | Protein phosphatase 1 regulatory subunit 12C and T-cell activation Rho GTPase-activating protein |
| **Molecular function:** | | | | | |
| GO:0005200 | structural constituent of cytoskeleton | 3 | 8.55E-04 | P02449, P02450 and Q9PSV3 | Feather keratin, Feather keratin 1 and Feather keratin Cos2-3 |

# Supplementary Table 6. GO enrichment analysis of expanded gene families of Asian king vulture.

| **Term** | **Description** | ***P*-value** | **Gene** | **Fold Enrichment** |
| --- | --- | --- | --- | --- |
| **Biological process:** | | | | |
| GO:0006953 | acute-phase response | 0.029 | *CD163* and *HFE* | 63.29 |
| GO:0002526 | acute inflammatory response | 0.081 | *CD163* and *HFE* | 21.66 |
| GO:0007156 | homophilic cell adhesion via plasma membrane adhesion molecules | 0.095 | *PCDHGA12* and *PCDHA1* | 18.44 |
| GO:1903827 | regulation of cellular protein localization | 0.048 | *ADCY10, HFE* and *CEP250* | 7.66 |
| GO:0007283 | spermatogenesis | 0.053 | *MAJIN, ADCY10* and *SPAG4* | 7.25 |
| GO:0048232 | male gamete generation | 0.054 | *MAJIN, ADCY10* and *SPAG4* | 7.20 |
| GO:0007276 | gamete generation | 0.078 | *MAJIN, ADCY10* and *SPAG4* | 5.85 |
| GO:0044703 | multi-organism reproductive process | 0.026 | *MAJIN, ADCY10, HFE* and *SPAG4* | 5.48 |
| GO:0022414 | reproductive process | 0.062 | *MAJIN, ADCY10, HFE* and *SPAG4* | 3.88 |
| GO:0000003 | reproduction | 0.062 | *MAJIN, ADCY10, HFE* and *SPAG4* | 3.87 |
| GO:0051641 | cellular localization | 0.092 | *MAJIN, ADCY10, HFE, CEP250* and *ANKS1B* | 2.53 |
| **Cellular component:** | | | | |
| GO:0031229 | intrinsic component of nuclear inner membrane | 0.011 | *MAJIN* and *SPAG4* | 170.44 |
| GO:0005639 | integral component of nuclear inner membrane | 0.011 | *MAJIN* and *SPAG4* | 170.44 |
| GO:0044453 | nuclear membrane part | 0.011 | *MAJIN* and *SPAG4* | 161.92 |
| GO:0045178 | basal part of cell | 0.049 | *ADCY10* and *HFE* | 36.39 |
| GO:0005637 | nuclear inner membrane | 0.054 | *MAJIN* and *SPAG4* | 32.71 |
| GO:0005887 | integral component of plasma membrane | 0.062 | *CD163, PCDHGA12, PCDHA1* and *HFE* | 3.82 |
| GO:0048471 | perinuclear region of cytoplasm | 0.068 | *ADCY10, HFE* and *CEP250* | 6.24 |
| GO:0005929 | cilium | 0.069 | *ADCY10, SPAG4* and *CEP250* | 6.17 |
| GO:0031226 | intrinsic component of plasma membrane | 0.070 | *CD163, PCDHGA12, PCDHA1* and *HFE* | 3.65 |

#

# Supplementary Table 7. A list of positively selected genes in the Asian king vulture.

| **Gene name** | **Description** | **dN/dS** | **FDR** | ***P*-Value** |
| --- | --- | --- | --- | --- |
| *HOXC9* | homeobox C9 | 45.24 | 0.000 | 8.65E-10 |
| *HPRT1* | hypoxanthine phosphoribosyltransferase 1 | 42.07 | 0.000 | 1.60E-11 |
| *TNFSF15* | TNF superfamily member 15 | 25.52 | 0.039 | 7.14E-03 |
| *SDSL* | serine dehydratase like | 21.71 | 0.031 | 5.41E-03 |
| *TPM2* | tropomyosin 2 | 18.60 | 0.000 | 6.23E-09 |
| *UTS2B* | urotensin 2B | 17.45 | 0.002 | 1.24E-04 |
| *ACAN* | aggrecan | 15.08 | 0.000 | 1.04E-14 |
| *TMPO* | thymopoietin | 14.05 | 0.039 | 7.34E-03 |
| *EIF4E3* | eukaryotic translation initiation factor 4E family member 3 | 12.36 | 0.006 | 3.94E-04 |
| *ARL10* | ADP ribosylation factor like GTPase 10 | 11.91 | 0.012 | 1.15E-03 |
| *PLPP3* | phospholipid phosphatase 3 | 10.94 | 0.018 | 1.88E-03 |
| *CYP1A2* | cytochrome P450 family 1 subfamily A member 2 | 10.91 | 0.027 | 4.13E-03 |
| *VPREB3* | V-set pre-B cell surrogate light chain 3 | 9.54 | 0.022 | 2.74E-03 |
| *GPR89A* | G protein-coupled receptor 89A | 9.16 | 0.000 | 4.63E-11 |
| *TRAPPC2L* | trafficking protein particle complex subunit 2L | 8.21 | 0.022 | 2.90E-03 |
| *SRM* | spermidine synthase | 7.85 | 0.000 | 3.26E-06 |
| *PDCD2* | programmed cell death 2 | 6.78 | 0.000 | 4.97E-09 |
| *TSPAN1* | tetraspanin 1 | 6.16 | 0.022 | 2.88E-03 |
| *ACTN1* | actinin alpha 1 | 5.59 | 0.000 | 5.11E-06 |
| *POLL* | DNA polymerase lambda | 4.48 | 0.002 | 9.38E-05 |
| *NIPSNAP2* | nipsnap homolog 2 | 4.40 | 0.013 | 1.30E-03 |
| *ACTN4* | actinin alpha 4 | 4.27 | 0.005 | 3.12E-04 |
| *BIRC5* | baculoviral IAP repeat containing 5 | 4.00 | 0.047 | 9.31E-03 |
| *CD274* | CD274 molecule | 3.99 | 0.031 | 5.34E-03 |
| *ZFAND6* | zinc finger AN1-type containing 6 | 3.92 | 0.022 | 2.52E-03 |
| *MRC1* | mannose receptor C-type 1 | 2.72 | 0.000 | 1.13E-05 |
| *TBCE* | tubulin folding cofactor E | 2.41 | 0.002 | 7.69E-05 |
| *AHR* | aryl hydrocarbon receptor | 2.32 | 0.003 | 1.46E-04 |
| *FUBP3* | far upstream element binding protein 3 | 2.16 | 0.009 | 7.97E-04 |
| *CNGA2* | cyclic nucleotide gated channel subunit alpha 2 | 2.14 | 0.022 | 3.18E-03 |
| *ZCCHC8* | zinc finger CCHC-type containing 8 | 2.03 | 0.037 | 6.55E-03 |
| *FRRS1* | ferric chelate reductase 1 | 1.83 | 0.046 | 9.04E-03 |
| *PROX1* | prospero homeobox 1 | 1.51 | 0.008 | 7.21E-04 |
| *RCSD1* | RCSD domain containing 1 | 1.47 | 0.018 | 1.93E-03 |
| *IFNLR1* | interferon lambda receptor 1 | 1.41 | 0.028 | 4.52E-03 |
| *SHPK* | sedoheptulokinase | 1.37 | 0.029 | 4.76E-03 |
| *CSPG4* | chondroitin sulfate proteoglycan 4 | 1.36 | 0.022 | 2.54E-03 |
| *TMEM171* | transmembrane protein 171 | 1.32 | 0.043 | 8.31E-03 |
| *MYOF* | myoferlin | 1.30 | 0.000 | 3.91E-10 |
| *SIM2* | SIM bHLH transcription factor 2 | 1.28 | 0.008 | 6.41E-04 |
| *EPB41L1* | erythrocyte membrane protein band 4.1 like 1 | 1.21 | 0.008 | 6.20E-04 |
| *ETF1* | eukaryotic translation termination factor 1 | 1.13 | 0.008 | 6.09E-04 |
| *CCDC191* | coiled-coil domain containing 191 | 1.04 | 0.022 | 3.01E-03 |
| *ACCS* | 1-aminocyclopropane-1-carboxylate synthase homolog | 1.02 | 0.031 | 5.22E-03 |

Abbreviation: FDR, False discovery rate

**Supplementary Table 8.** Functional enrichment of positively selected genes in Asian king vulture.

| **Term** | **Description** | ***P*-Value** | **Gene** | **Fold Enrichment** |
| --- | --- | --- | --- | --- |
| **Biological process:** | | | | |
| GO:1901978 | positive regulation of cell cycle checkpoint | 0.04 | *BIRC5* and *PROX1* | 53.80 |
| GO:0002837 | regulation of immune response to tumor cell | 0.04 | *CD274* and *AHR* | 44.02 |
| GO:0002834 | regulation of response to tumor cell | 0.05 | *CD274* and *AHR* | 40.35 |
| GO:0002418 | immune response to tumor cell | 0.06 | *CD274* and *AHR* | 33.39 |
| GO:0071353 | cellular response to interleukin-4 | 0.07 | *SHPK* and *MRC1* | 27.67 |
| GO:0070670 | response to interleukin-4 | 0.07 | *SHPK* and *MRC1* | 26.17 |
| GO:0002347 | response to tumor cell | 0.09 | *CD274* and *AHR* | 20.17 |
| GO:1901976 | regulation of cell cycle checkpoint | 0.09 | *BIRC5* and *PROX1* | 19.76 |
| GO:0072091 | regulation of stem cell proliferation | 0.03 | *ACAN, PDCD2* and *PROX1* | 10.53 |
| GO:0055001 | muscle cell development | 0.01 | *ACTN1, MYOF, ACTN4* and *PROX1* | 9.73 |
| GO:0002831 | regulation of response to biotic stimulus | 0.05 | *CD274, IFNLR1* and *AHR* | 7.89 |
| GO:0072089 | stem cell proliferation | 0.05 | *ACAN, PDCD2* and *PROX1* | 7.73 |
| GO:0071219 | cellular response to molecule of bacterial origin | 0.01 | *CD274, SHPK, MRC1* and *AHR* | 7.69 |
| GO:0030324 | lung development | 0.06 | *CYP1A2, PROX1* and *SIM2* | 7.49 |
| GO:0030323 | respiratory tube development | 0.06 | *CYP1A2, PROX1* and *SIM2* | 7.34 |
| GO:0043281 | regulation of cysteine-type endopeptidase activity involved in apoptotic process | 0.06 | *TNFSF15, BIRC5* and *PDCD2* | 7.26 |
| GO:0071216 | cellular response to biotic stimulus | 0.02 | *CD274, SHPK, MRC1* and *AHR* | 6.84 |
| GO:0060541 | respiratory system development | 0.07 | *CYP1A2, PROX1* and *SIM2* | 6.60 |
| GO:0097164 | ammonium ion metabolic process | 0.07 | *HPRT1, PLPP3* and *SRM* | 6.57 |
| GO:2000116 | regulation of cysteine-type endopeptidase activity | 0.08 | *TNFSF15, BIRC5* and *PDCD2* | 6.21 |
| GO:0071222 | cellular response to lipopolysaccharide | 0.08 | *CD274, SHPK* and *MRC1* | 6.13 |
| GO:0002237 | response to molecule of bacterial origin | 0.04 | *CD274, SHPK, MRC1* and *AHR* | 4.90 |
| GO:1901615 | organic hydroxy compound metabolic process | 0.02 | *SHPK, CYP1A2, HPRT1, PROX1* and *PLPP3* | 4.35 |
| GO:1901361 | organic cyclic compound catabolic process | 0.06 | *ZCCHC8, CYP1A2, ETF1* and *HPRT1* | 4.30 |
| GO:0003012 | muscle system process | 0.07 | *TPM2, TBCE, MYOF* and *RCSD1* | 4.09 |
| GO:0042692 | muscle cell differentiation | 0.08 | *ACTN1, MYOF, ACTN4* and *PROX1* | 3.91 |
| GO:0034097 | response to cytokine | 0.02 | *CD274, SHPK, ZFAND6, MRC1, IFNLR1, ACTN4* and *SRM* | 3.28 |
| GO:0030036 | actin cytoskeleton organization | 0.06 | *EPB41L1, TPM2, ACTN1, ACTN4* and *PROX1* | 3.21 |
| GO:0071345 | cellular response to cytokine stimulus | 0.04 | *SHPK, ZFAND6, MRC1, IFNLR1, ACTN4* and *SRM* | 3.13 |
| GO:0000904 | cell morphogenesis involved in differentiation | 0.07 | *ACTN1, TBCE, ACTN4, HPRT1* and *PROX1* | 3.06 |
| GO:0030029 | actin filament-based process | 0.10 | *EPB41L1, TPM2, ACTN1, ACTN4* and *PROX1* | 2.78 |
| GO:0007010 | cytoskeleton organization | 0.10 | *EPB41L1, TPM2, ACTN1, TBCE, BIRC5, ACTN4* and *PROX1* | 2.13 |
| GO:0008283 | cell proliferation | 0.05 | *ACAN, CD274, BIRC5, IFNLR1, CSPG4, PDCD2, HPRT1, AHR* and *PROX1* | 2.08 |
| **Cellular component:** | | | | |
| GO:0031143 | pseudopodium | 0.04 | *ACTN1* and *ACTN4* | 50.74 |
| GO:0005884 | actin filament | 0.03 | *TPM2, ACTN1* and *RCSD1* | 10.70 |
| GO:0030017 | sarcomere | 0.08 | *TPM2, ACTN1* and *ACTN4* | 6.09 |
| GO:0044449 | contractile fiber part | 0.09 | *TPM2, ACTN1* and *ACTN4* | 5.68 |
| GO:0030016 | myofibril | 0.10 | *TPM2, ACTN1* and *ACTN4* | 5.52 |
| GO:0015629 | actin cytoskeleton | 0.03 | *CD274, TPM2, ACTN1, ACTN4* and *RCSD1* | 4.18 |
| GO:0099513 | polymeric cytoskeletal fiber | 0.10 | *TPM2, ACTN1, TBCE, BIRC5* and *RCSD1* | 2.76 |
| GO:0044431 | Golgi apparatus part | 0.07 | *ACAN, GPR89A, TRAPPC2L, CNGA2, CSPG4* and *PLPP3* | 2.57 |
| GO:0099512 | supramolecular fiber | 0.07 | *TPM2, ACTN1, TBCE, BIRC5, ACTN4* and *RCSD1* | 2.57 |
| GO:0099081 | supramolecular polymer | 0.08 | *TPM2, ACTN1, TBCE, BIRC5, ACTN4* and *RCSD1* | 2.54 |
| GO:0099080 | supramolecular complex | 0.08 | *TPM2, ACTN1, TBCE, BIRC5, ACTN4* and *RCSD1* | 2.54 |
| GO:0044433 | cytoplasmic vesicle part | 0.06 | *CD274, GPR89A, TRAPPC2L, ACTN1, MRC1, MYOF, CNGA2* and *ACTN4* | 2.19 |
| **Molecular function:** | | | | |
| GO:0016922 | ligand-dependent nuclear receptor binding | 0.09 | *ACTN4* and *PROX1* | 19.82 |
| GO:0005178 | integrin binding | 0.05 | *ACTN1, ACTN4* and *PLPP3* | 8.07 |
| GO:0051015 | actin filament binding | 0.02 | *TPM2, ACTN1, ACTN4* and *RCSD1* | 7.48 |
| GO:0003779 | actin binding | 0.02 | *EPB41L1, TPM2, ACTN1, ACTN4* and *RCSD1* | 4.64 |
| GO:0046982 | protein heterodimerization activity | 0.05 | *TPM2, BIRC5, AHR* and *SIM2* | 4.58 |
| GO:0042803 | protein homodimerization activity | 0.02 | *TPM2, ACTN1, BIRC5, ACTN4, AHR* and *SRM* | 3.49 |
| GO:0008092 | cytoskeletal protein binding | 0.03 | *EPB41L1, TPM2, ACTN1, TBCE, BIRC5, ACTN4* and *RCSD1* | 2.90 |
| GO:0032403 | protein complex binding | 0.09 | *TPM2, ACTN1, ACTN4, RCSD1* and *PLPP3* | 2.88 |
| GO:0046983 | protein dimerization activity | 0.04 | *TPM2, ACTN1, BIRC5, ACTN4, AHR, SIM2* and *SRM* | 2.66 |
| GO:0044877 | macromolecular complex binding | 0.08 | *TPM2, ACTN1, CNGA2, ETF1, ACTN4, RCSD1* and *PLPP3* | 2.22 |
| GO:1901363 | heterocyclic compound binding | 0.05 | *ZCCHC8, ARL10, EIF4E3, ZFAND6, ACTN1, AHR, ACTN4, PROX1, ACCS, SDSL, FUBP3, SHPK, CYP1A2, CNGA2, PDCD2, POLL, ETF1, HPRT1, HOXC9, SIM2* and *TMPO* | 1.41 |
| GO:0097159 | organic cyclic compound binding | 0.06 | *ZCCHC8, ARL10, EIF4E3, ZFAND6, ACTN1, AHR, ACTN4, PROX1, ACCS, SDSL, FUBP3, SHPK, CYP1A2, CNGA2, PDCD2, POLL, ETF1, HPRT1, HOXC9, SIM2* and *TMPO* | 1.39 |

**Supplementary Table 9.** Positively selected genes with deleterious amino acid substitutions, compared to the red junglefowl reference genome.

| **Gene** | **Amino acid substitution** | **PROVEAN score** |
| --- | --- | --- |
| 1-aminocyclopropane-1-carboxylate synthase homolog (*ACCS*) | E226W | -5.75 |
| actinin alpha 4 (*ACTN4*) | A243L | -2.742 |
| actinin alpha 4 (*ACTN4*) | N241G | -3.341 |
| aggrecan (*ACAN*) | V41A | -2.633 |
| aggrecan (*ACAN*) | V1308A | -2.936 |
| aggrecan (*ACAN*) | G1392K | -3.342 |
| aggrecan (*ACAN*) | G934S | -3.908 |
| aggrecan (*ACAN*) | H1974Y | -3.936 |
| aggrecan (*ACAN*) | D24G | -4.582 |
| aggrecan (*ACAN*) | F623I | -5.8 |
| aryl hydrocarbon receptor (*AHR*) | D81L | -4.336 |
| baculoviral IAP repeat containing 5 (*BIRC5*) | P56R | -7.307 |
| CD274 molecule (*CD274*) | Y164F | -2.759 |
| chondroitin sulfate proteoglycan 4 (*CSPG4*) | E890L | -3.002 |
| cytochrome P450 family 1 subfamily A member 2 (*CYP1A2*) | L342V | -2.627 |
| cytochrome P450 family 1 subfamily A member 2 (*CYP1A2*) | V343A | -3.423 |
| cytochrome P450 family 1 subfamily A member 2 (*CYP1A2*) | T394A | -4.246 |
| eukaryotic translation initiation factor 4E family member 3 (*EIF4E3*) | K188L | -5.39 |
| eukaryotic translation initiation factor 4E family member 3 (*EIF4E3*) | Y187S | -6.887 |
| ferric chelate reductase 1 (*FRRS1*) | P410L | -3.31 |
| ferric chelate reductase 1 (*FRRS1*) | G82H | -4.801 |
| G protein-coupled receptor 89A (*GPR89A*) | S13K | -2.714 |
| homeobox C9 (*HOXC9*) | R229H | -2.968 |
| homeobox C9 (*HOXC9*) | M252E | -3.102 |
| homeobox C9 (*HOXC9*) | M249W | -3.223 |
| homeobox C9 (*HOXC9*) | N232G | -4.046 |
| homeobox C9 (*HOXC9*) | M217P | -4.631 |
| homeobox C9 (*HOXC9*) | D222K | -4.946 |
| homeobox C9 (*HOXC9*) | Y225I | -5.3 |
| hypoxanthine phosphoribosyltransferase 1 (*HPRT1*) | E130Q | -2.687 |
| hypoxanthine phosphoribosyltransferase 1 (*HPRT1*) | V129F | -4.325 |
| hypoxanthine phosphoribosyltransferase 1 (*HPRT1*) | V126F | -4.42 |
| hypoxanthine phosphoribosyltransferase 1 (*HPRT1*) | L127Y | -4.484 |
| hypoxanthine phosphoribosyltransferase 1 (*HPRT1*) | N125F | -7.832 |
| mannose receptor C-type 1 (*MRC1*) | T799A | -3.348 |
| mannose receptor C-type 1 (*MRC1*) | G371S | -3.474 |
| myoferlin (*MYOF*) | E1443F | -3.445 |
| nipsnap homolog 2 (*NIPSNAP2*) | E150A | -2.687 |
| phospholipid phosphatase 3 (*PLPP3*) | C43S | -7.486 |
| serine dehydratase like (SDSL) | T96A | -4.153 |
| SIM bHLH transcription factor 2 (*SIM2*) | Q152S | -3.172 |
| spermidine synthase (*SRM*) | L291S | -4.599 |
| urotensin 2B (*UTS2B*) | A119G | -2.5 |
| urotensin 2B (*UTS2B*) | C120R | -9.281 |
